# “ *Blaming, shaming, humiliation*”: Stigmatising medical interactions among people with non-epileptic seizures

**DOI:** 10.12688/wellcomeopenres.12133.2

**Published:** 2017-10-24

**Authors:** Catherine Robson, Olaug S. Lian

**Affiliations:** 1 Department of Research Capacity Development, Nelson Mandela University, Port Elizabeth, South Africa; 2 Department of Community Medicine, Faculty of Health Sciences, University of Tromsø – The Arctic University of Norway, Tromsø, Norway

**Keywords:** psychogenic non-epileptic seizures, non-epileptic attack disorder, medically unexplained symptoms, doctor-patient interactions, patient experience, stigma

## Abstract

**Background**: People with non-epileptic seizures (NES) describe challenging relationships with health professionals, and explain negative interactions as common and expected. Despite these difficulties, little is known about how people with NES experience difficult healthcare encounters.

**Methods**: Using a thematic discourse analysis approach, we analysed the free-text survey responses of 135 people with NES and asked: what kind of challenges do people living with this condition encounter when interacting with health professionals, and how do they experience the consequences of difficult interactions? We explore their experiences by interpreting the latent meaning of participants’ texts from a social-constructionist perspective on health and illness.

**Results**: The overarching narrative depicts a fundamental breakdown in patient-provider relationships. According to our data, the negative experiences of study participants emerge from more than practitioners’ lack of awareness of NES and access to information about the condition - to the extent that it is available. In examining the challenges people with NES encounter when interacting with health professionals, their main experiences centre on blame and humiliation. When exploring their experiences, theories of stigma serve as a useful theoretical framework.

**Conclusions**: Normative judgements arising from psychogenic understandings of NES are stigmatising and restrict professional displays of respectful (patient-centred) care. Those with the condition depict being negatively stereotyped, illegitimated and held morally culpable by health professionals. Perceived to lack medical, moral and credible status, participants describe practitioners who treat them with disrespect, and some recount conduct that defies all ethical and professional obligations and standards. These encounters can have wide-ranging adverse consequences for patients: emotionally, physically, and for their future healthcare. The quality of healthcare interactions for people with NES requires urgent improvements. In addition to increased awareness of the condition, practitioners need to be conscious of making and acting on adverse moral appraisals when interacting with this patient group.

## Introduction

Patient experience is recognised as a crucial dimension of health-care quality, and is shown to have important implications for patient safety, health outcomes, and resource use ^1^. In Western healthcare systems, there is a mandate to report on the metrics of patient experience ^2^, underscored by a raft of policy initiatives that emphasise the importance of person/patient-centred care ^3^.

Within patient narratives, the voice of people with medically unexplained seizures, usually medically labelled psychogenic non-epileptic seizures (PNES) or non-epileptic attack disorder (NEAD) – is practically absent. We refer to the seizures and condition using the shorthand term, non-epileptic seizures (NES). A recent systematic synthesis of the qualitative literature identified just 21 studies exploring insights into experiences of living with the condition ^4^; predominantly comprised of small-scale interview and interactional studies.

NES are defined by their resemblance to epileptic seizures, but unlike epilepsy, are not considered to be caused by abnormal electrical discharges in the brain. The condition is characterised as psychogenic and classified under the banner of somatoform, functional, and dissociation or conversion disorder - depending on the clinician and taxonomy consulted. In these hypothetical models, maladaptive psychological functioning (dysfunctional emotional and coping mechanisms) give rise to physical symptoms (seizures); which are involuntary and outside of the patients’ control ^5^.

Most patients with NES are initially thought to have epilepsy, and it typically takes several years for the correct diagnosis to be made ^6^. Prevalence is estimated as 2 to 33 cases per 100,000 of the general population (on par with multiple scoliosis) ^7^. Approximately 30% of cases seen in specialist seizure centres are related to NES ^8^, and around 75% of people diagnosed with the condition are women ^9^. The preferred treatment is psychotherapy. A recent Cochrane review concluded that therapeutic services are hard to secure, often time limited, adherence and success rates are modest, and there is little reliable evidence to support the use of any treatment for the condition ^10^. 70% of people with NES are thought to remain disabled several years after the diagnosis ^11^.

Health professionals across disciplines find interactions with NES patients “tough”, “uncomfortable”, “challenging” and “frustrating” ^12– 16^. Likewise, people with NES describe difficult and challenging relationships with clinicians ^17, 18^, and explain negative experiences as common and expected ^4, 19– 21^. Despite these difficulties, little is known about how negative healthcare interactions are experienced by people with NES. Patients' perceptions of negative experiences are an important source of information about the quality of health care received ^22^, and can supply the evidence needed for change ^23^.

This qualitative study seeks to fill gaps in our knowledge by exploring the free-text responses of 135 people with NES. Our main aim is to explore implications of challenging encounters with health professionals for people with NES. Using a thematic discourse analysis approach, we ask: How do people with NES experience negative interactions with health professionals, what kind of challenges do they encounter, and how do they experience the consequences of difficult healthcare interactions?

## Methods

A link to an in-depth (86-item) survey was advertised to members of 20 patient and practitioner-led online support groups for people with NES (not disclosed for reasons of confidentiality). The overall aim of the study was to investigate the healthcare experiences of people living with NES, explore how challenging clinical encounters affect patients, and give voice to those living with the condition. The wider research project aims to examine the diagnostic journey of people with NES, identify forms of support and treatment that are most helpful to people living with the condition, and to explore the social impact of having NES. Final survey data were organized around four key themes: 1) the diagnostic journey 2) access to and experience of treatment 3) interactions with healthcare professionals and 4) social support and social stigma. By allowing their voices to be heard, we hope NES patient narratives will help health care professionals better understand the experiences and perceptions of those they examine, diagnose, and treat.

To include as many people with NES as possible, the only inclusion criteria were that participants were over 18 years of age and had received a diagnosis of NES by a health professional. Participants were advised that we use the term NES to describe diagnoses of psychogenic non-epileptic seizures (PNES), non-epileptic attack disorder (NEAD), and other diagnostic terms sometimes used to describe the condition and symptoms, such as, dissociative, conversion, functional, and pseudo seizures. Participants were able to save their answers and return to the survey via a secure and automated email link. The smart-logic survey format helped to protect against participants giving conflicting answers, and to ‘re-check’ and correct responses when they did so. The survey was piloted among 25 people living with the condition. Advertising commenced May 2016 and final data were collected from 1 July to 1 October 2016.

### Participants

141 participants completed all mandatory questions and submitted their responses for inclusion in the study. Six people reported a diagnosis other than NES (functional movement disorder), and their responses were excluded from further analysis. The socio-demographic and health characteristics of the 135 participants on which we report are presented in Table 1.

**Table 1.** Socio-demographic and health characteristics of the study participants.

		**Number or** **median**	**Proportion** **or range**
Country of residence	UK (69) and Ireland (3)	72	53%
	US (54) and Canada (3)	57	42%
	Rest of the world (Australia, New Zealand and Norway)	6	4%
Age	All	38 years	18 – 73 years
	18 to 30 years	42	31%
	31 to 45 years	53	39%
	46 to 59 years	35	26%
	60+ years	5	4%
Gender	Female	118	87%
	Male	14	10%
	Transgender	3	2%
Relationship status	Married or Civil Union (64) or partnered (19)	83	61%
	Single (38), separated or divorced (13) or widowed (1)	52	39%
Employment status	In full-time (15) or part-time employment (19) or education (9)	43	32%
	Unable to work (87) or looking for work (1)	88	65%
	Retired	4	3%
Diagnostic status	A formal (certain or highly likely) diagnosis of NES	115	85%
	A tentative (likely or fairly likely) diagnosis of NES	20	15%
Diagnosis of NES received from*	A neurologist who specialises in seizures	89	66%
	A neurologist who does not specialise in seizures	36	28%
	A psychiatrist or psychologist	31	23%
Time from onset	All	4 to 5 years	<1 year to 20+ years
Prior erroneous diagnosis of epilepsy+	Yes	32	24%
	No	99	76%
Dual seizure diagnosis	NES alone	116	86%
	Epilepsy and NES	19	14%

Key: * Multiple answers possible ^ Categories capped at 100+ + 4 cases missing

### Data

**Figure 1.**
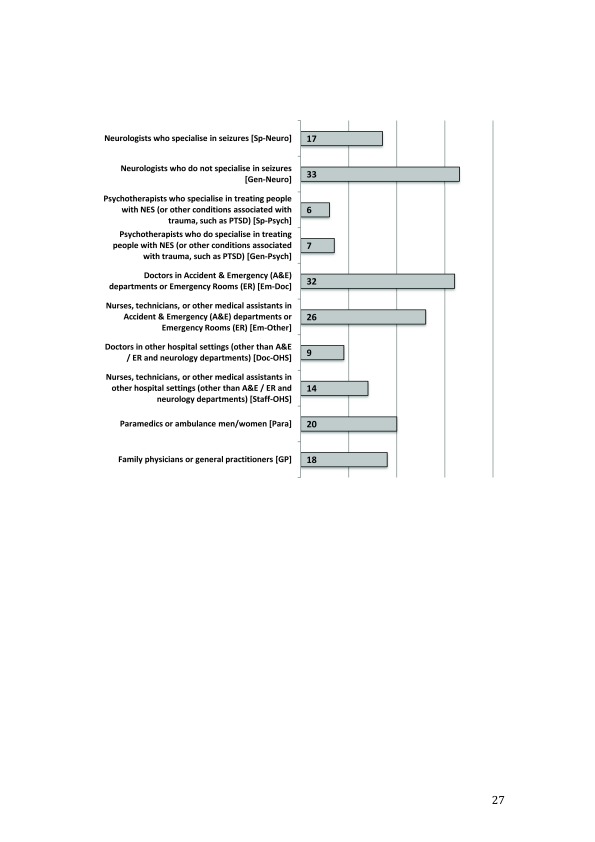
Type of health professional(s) involved in participants’ descriptions of their ‘single worst’ health care interaction relating to NES.

Qualitative data is derived from responses to two open-text questions, from theme 3 of the survey, “interactions with healthcare professionals”. Participants were asked to “please think about the single worst interaction you have had with a health professional about NES. Choosing this question means that we explicitly asked participants to recount negative experiences, and the results must be interpreted accordingly. In connection to this question participants were asked to select the type of health professional involved in the interaction from a list of 11 professions (detailed in Figure 1). In eight cases, participants chose the option ‘other’, and described the type of profession discussed. Following a review, the health professionals indicated were amalgamated in pre-determined categories (e.g. ‘family physician at hospital’ was coded under ‘GP’). Participants could indicate if more than one type of health professional was present in the interaction, and 47 of them did so. Participants were then instructed (the question was mandatory) to “please describe what you disliked about the interaction”. Later in the survey, participants were invited to answer an optional question: “In relation to NES, is there anything else you would like to tell us about your experiences of interacting with health professionals?” To which, 69 participants in our sample responded.

### Analytical approach

Qualitative data were gathered in one document and anonymised. Taken together, texts amounted to around 12,000 words (approximately 90 words per respondent). Themes related to our research questions were generated inductively from the data using a thematic discourse analysis approach ^24^. Seeking to explore the reality of participants’ negative experiences (as they express it), we initially employed an essentialist approach to the analysis of their texts – maintaining a semantic focus on the topical content of respondents’ descriptions.

In the first phase of analysis, we worked in conjunction to identify commonly recurring patterns present in participants’ descriptions of interactions. In the second phase of analysis, we reviewed provisional key themes for consistency, continuously discussing and revising our interpretations and classifications. Settling on six key themes, we coded the data first individually, then both of us together. During this phase, we re-checked our readings several times. In the final round, we identified and resolved (minimal) competing interpretations.

We present illustrative extracts related to our main themes, limiting ourselves to two to three quotes per sub-section. Because themes are partly overlapping, and many text-sections relate to more than one theme, they could be structured differently. ID refers to participants’ unique identifying number, and the type(s) of health professional(s) to which they refer (as detailed in Figure 1) are indicated. An additional category (‘all health professionals’: All-HP) was created to signify responses from the optional question, to which participants typically responded by describing experiences with health care professionals in general (e.g. using the terms “all of them”, “doctors”, “they”).

The purpose of the data analysis was to seek an interpretative understanding of participant’s stories ^25^, which means that they “hold truth in the sense that they allow us to understand reality from the point of view of the person expressing these comments” ^26^. In line with this perspective, we do not question whether expressed experiences are right or wrong, good or bad. Although our interpretation of the data is qualitative, we try to separate the most frequent experiences from those less frequent by adding words like "frequent", "often" and "typically".

In the discussion, we consider findings in relation to previous literature and offer interpretation of their latent meaning from a social-constructionist perspective on health and illness, using the concept of stigma.

## Results

### Negative and disrespectful encounters

Participants’ texts suggest negative healthcare encounters are common. Several describe identifying their ‘worst’ healthcare interaction in relation to NES as a struggle (e.g. “ *this is hard as there have been a lot*”, “ *so many horrid experiences”,* and *“difficult to choose”*).

Overwhelmingly, participants who answered the optional question (“ …is there anything else you would like to tell us about your experiences of interacting with health professionals?”) responded by describing negative experiences with health care professionals or expressing dissatisfaction with medical culture in general.

Many explicitly (and spontaneously) describe negative interactions with health professionals as typical, and the norm:

*“I can only say that I have had far far more negative experiences with supposed medical professionals in relation to NES, than positive experiences”* (ID43726078:All-HP).
*“You only get a small few who actually understand the condition and treat you well […] then you get those that will just think you are faking and a waste of time, and they go out of their way to make you feel like that. […] I think it is disgraceful”* (ID44862903:All-HP).
*“All of them. All interactions have been negative with blaming, shaming, humiliation, and emotional pain”* (ID43349947:All-HP).

Participants’ narratives are characterised by encountering health professionals who they perceive to display or who demonstrate disrespectful (e.g. “ *offensive*”, “ *rude*”, “ *antagonistic*”, “ *degrading*”, “ *derogatory*”) attitudes and behaviours:

*“Doctors in general […] have been dismissive, rude and talked about me as if I wasn't there”* (ID41116112:All-HP).
*“… I heard the paramedics discussing […] "Yeah, I don't want to explain how you lost [the medication] and she's faking anyway." From there I don't recall exact words, but they went on to degrade me as a person. They were wheeling me into the ER as they were continuing their derogatory conversation, the ER staff joined in …”* (ID40936008:Para:Em-Doc:Em-other).
*“The professional I met was very rude and offensive to my situation and sort of threw me out of his office once he found that my situation was a hopeless one as far as his medical expertise was concerned. I felt that he was making sure that I felt I had wasted his time […] I was told that I had "attacks" and that what I was experiencing were NOT seizures at all”* (ID40440707:Sp-Neuro).

Participants explain that they find these types of encounters “ *appalling*”, “ *offensive*”, “ *disgraceful*” and “ *shocking*”:

*“They were rude and isolated me. They accused me of doing recreational drugs and that I was intentionally doing the seizures […] I was even accused of 'seeking attention'. I was discriminated against and their attitude was appalling”* (ID42582634:Em-Doc:Em-other).
*“…I recall coming out of a seizure at one point and hearing one of the paramedics say to his partner, ‘I really think she's just faking this’. […] I was not able to respond […] completely horrified and shocked that someone would even think such a thing, let alone voice the words out loud with the patient laying inches from you”* (ID43726078:Para).

### Lack of knowledge and awareness

Participants most often (directly) attribute negative interactions and disrespectful professional attitudes and behaviours to a lack of knowledge and awareness of the condition:

*“A lot of professionals are unaware of the condition so therefore are quite dismissive and either don't believe you or think it's nothing/we fake it”* (ID40934620:All-HP).
*“There needs to be more knowledge out there for medical professionals. They are here to help us, not traumatize us”* (ID41819554:All-HP).
*“So many health professionals understand very little about the condition, and therefore treatment/interactions can seem/be very unsatisfactory”* (ID40933009:All-HP).

More frequently, they describe a lack of practitioner awareness and understanding of NES in relation to specific adverse events:

*“He laughed in my face at the diagnosis of FND [Functional Neurological Disorder] and NEAD and said ‘what’s that’. I realised I knew more than he did about my problems. I don't see him anymore”* (ID41429984:GP).
*“… when my husband objected to them injecting me with medicine they called the police […] My husband tried to explain […] the medication that he was fixing to inject into me would do nothing to help my disorder. I finally came to and told them the same thing. They had never heard of such a thing”* (ID43728373:Para).

Lack of practitioner awareness and understanding, they often explain, is prevalent among the medical community:

*“Many do not have a clue”* (ID41780739:All-HP).
*“None of them listen […] or can even tell you what a non-epileptic seizure is […] there seems a chronic ignorance”* (ID41454126:All-HP).

Who, participants typically describe, are unsure how to work with them:

*“My GP does not seem to understand what is going on and every time I go and ask for help or advice I get nothing from her”* (ID40151428:GP).
*“They see "pseudoseizures" on my chart and avoid me like I am an axe murderer”* (ID41489268:All-HP).
*“At GP level they know little or nothing, even some neurologist run from you …”* (ID41429984:All-HP).

### Illegitimate patients

Our participants frequently explain that the condition is “ *not taken seriously*”, and describe a lack of illness recognition among health professionals:

*“All around, the places and people I have checked off treat PNES as if it were an imaginary friend. Fake, irrational, and made up to seek attention. It is not taken seriously and therefore finding help is nearly impossible”* (ID42477133:All-HP).
*“Not taken seriously although it changed my whole life”* (ID41453407:All-HP).
*“I feel that I don’t get taken seriously enough and that nobody cares. I am made to feel like I am faking the seizures, which I find extremely disheartening as how anyone can fake them is beyond me. It's no life for anyone”* (ID40981734:All-HP).

They often report being told by health professionals that there is “ *nothing [physically] wrong*” with them and they have “ *no medical problem*”:

*“He simply said they could find no medical problem, I would probably never have another seizure, and he didn't want to see me in clinic and walked away from the bed. No help or advice given”* (ID41318794:Sp-Neuro).
*“Told that there is nothing wrong with me”* (ID40949276:Gen-Neuro).
*“… I was told I am not sick I am healthy and there is nothing wrong with me or my brain”* (ID41492322:GP).

Several describe meeting with health professionals who do not believe in the existence of the illness itself:

*“I was pretty much told that my condition didn’t really exist and that I was just hysterical and an attention seeker”* (ID41116112:Staff-OHS).
*“… this doctor seemed to not believe that NEAD existed”* (ID40949282:Em-Doc).
*“I haven't met a single one who believes this is an illness”* (ID41163538:All-HP).

The lack of legitimacy, some participants explain, is due to the lack of bio-markers for the condition:

*“… if neurologists don’t see it in a scan it doesn't exist”* (ID44796812:Gen-Neuro).

Many describe interactions in which a lack of positive biomarkers had been used to dismiss or diminish their symptoms or situation:

*“…when tests showed that I did not have epilepsy she was totally dismissive and rude she said there is nothing I can do to help you”* (ID40952225:Sp-Neuro).
*“…doctor in the hospital said that because there were no abnormalities in my brain waves that it could be nothing else but voluntary”* (ID43714636:Gen-Neuro)
*“The way he looked at me like I was crazy […] basically telling me it was all in my head by saying 'now you no nothing has shown up on your scans they will stop'. Never listened to me […] totally dismissed”* (ID44796812:Gen-Neuro).

They also depict practitioners who appear unwilling to investigate their ailments, and who attribute complaints to NES:

*“Not very interested if I go with something …. It’s to do with your NEAD”* (ID40950884:GP).
*“Doctor does not know what it is, get fed up with telling them. If I go with ailments get told its NEAD”* (ID44584667:GP).
*“My GP blamed my ear and sinus pain on my condition, even though after prompting her to look in my ears she found that I had a sinus and ear infection […] Even when visiting my dentist with tooth pain I was asked whether it could be due to clenching my jaw together when I am anxious!”* (ID41489268:All-HP).

Within their accounts, participants often report perceiving or being told that they are “ *time-wasters*”:

*“The neurologist told me I didn't have epilepsy […] NES wasn't explained […] I was made to feel like I had wasted their time, and that nothing was wrong with me”* (ID40932805:SP-Neuro)
*“Nurse I will never forget […] didn't understand the condition and was down right rude. When coming around from a seizure I can remember her just standing over me with her arms crossed just shouting "get up you are wasting my time, why do I have to put up with patients like you" (ID44862903:Staff-OHS).*
*“Told […] to go home and stop wasting time and money”* (ID40935644:Gen Neuro)

### Disregard of patients’ experiential perspectives

Our participants depict health professionals who they perceive not to “ *listen*” and who disregard or marginalise their subjective knowledge and experiences:

*“She didn't know what the heck was going on with me and chose not to even listen to me”* (ID42353316:Gen-Neuro).
*“The neurologist was more interested in my migraines than what was troubling me. He dismissed my concerns and just said they were "funny turns" and would go away eventually by themselves”* (ID44584911:Gen-Neuro).
*“…When I did come out of the seizure they were uninterested in hearing my side of the story and continued to believe I was a fake and tell the doctors at the ER that upon arrival” (ID41489268:Em-Doc:Para).*

Within their accounts, they describe practitioners who have little or no understanding of their situation:

*“…The doctor seemed not to understand my worries”* (ID44033148:GP).
*“… Terrible communication skills did not understand me at all”* (ID44727734:Em-Doc).
*“I don't feel as if any of my neurologists understand the human cost of this illness”* (ID41694668:All-HP).

The lack of understanding, participants explain, is often accompanied by a lack of empathy, compassion and support:

*“… so frustrating as they had no compassion or understanding and made me feel even worse about my condition”* (ID41575187:Staff-OHS).
*“NES/NEAD can severely disrupt lives, and there is not always enough understanding or support from professionals”* (ID40933009:All-HP).

Some participants describe health professionals who ignore or dismiss their expertise, and appear unwilling to learn more about the condition:

*“I find the majority of all in these fields don't care or want to learn about PNES”* (ID40179369:All-HP).
*“A lot of them don't know or don't want to know, sort of treat you as a crazy person”* (ID41014474:All-HP).
*“Really belittled the that fact that it was really a thing […] He knows best and there is no way, even after tons of research, that we know more than him”* (ID40951076:GP).

### Voluntary control

Several participants report encountering health professionals whom they perceive to hold them accountable for onset of the illness:

*“They made me feel like it was my fault. That I somehow over-stressed myself to the point that I couldn’t just handle it. So my body was reacting with these "fake" pseudoseizures. In other words it was all in my head”.* (ID43726369:Gen-Neuro)
*“He was not interested in me as I only had NES and not epilepsy and that I brought it on and can control it, which I can’t”* (ID43511449:Gen-Neuro).

More frequently, participants describe being caught in encounters in which it is suspected they have voluntarily, conscious control:

*“Told me I am going crazy and I have conscious control over everything and nothing is wrong I am just lazy and go to the hospital frequently because I have nothing better to do”* (ID41066720:Em-Doc).
*“The fact that they were rude, implied that I […] had control over the seizures, left me in urine soaked clothes after I had lost continence during a seizure, left me unattended during an event with the bed railing down I fell out and blacked my eye and temple”* (ID43737972:Gen-Neuro:Doc-OHS).
*“… He was so rude tried implying it was all in my head then showed me correspondence from my neurologist who had put she is suspicious that I am putting them on”* (ID40142452:Gen-Neuro:GP).

A major feature of participants’ narratives is meeting health professionals who explicitly accuse them of being a “ *faker*”:

*“An ER doctor threw water on my face when he thought I was mid-episode […] I flinched, he loudly proclaimed, ‘She's a faker. Discharge her’”* (ID41070264:Em-Doc:para).
*“[…] I heard a nurse come in and say ‘ [name], enough of the faking it’ […] She yelled out ‘if you are having a seizure I can open your eyes you are faking it’ […] she walked away saying ‘give me a break what a faker’”* (ID40969892:Em-OS).
*“The doctor told me I was faking. He stabbed my arms with a needle whilst I was paralyzed to prove I was faking […] He kept telling me I was faking and there is nothing wrong with me”* (ID41581556:Em-Doc).

Many participants recollect being accused of feigning the seizures for attention:

*“I was told everyone was gone out of the room, the drama can stop. That I only do this for attention seeking”* (ID40933181:Em-Doc).
*“They told me to my face that I was making it up and I am doing it myself for attention”* (ID41441665:Para).
*“I was accused of faking a seizure […] was looking for attention […] told to pull myself together and go home”* (ID44095893:GP)

Others describe encountering health professionals who they perceive had accused them of malingering (“faking” for secondary material gain):

*“The neurologist implied that I was faking them to get out of work”* (ID44768456:Gen-Neuro).
*“Believed I had been faking it to further my work cover claim”* (ID43738023:Gen-Neuro).
*“Told I was faking it because I must be a drug addict trying to score drugs”* (ID40935644:Gen-Neuro).

These perceptions, participants describe, contribute to why some health professionals appear unwilling to assess or treat seizure injuries:

*“I had fallen and hurt my shoulder and I couldn't move it. They refused to take me to A&E because they said that would be giving in to the attention that I wanted”* (ID41441665:Para).
*“He kept referring to NES as 'your kind of seizures' making me think he didn't believe. He didn't check me over at all because 'there was nothing wrong with me'. They were his actual words, so he had pre-judged me by what he had read on my notes. Even though I had actually damaged my arm. So because of my NEAD I got dismissed that day”* (ID40949282:Em-Doc).
*“Anytime I am in the emergency room I am treated like some kind of drug addict or attention seeker. Despite the fact I did not want to arrive in their ER that day and have a condition that can cause real injuries I am treated with disrespect […] I do not typically get evaluated, and am usually immediately discharged without the doctor even checking for injuries”* (ID41489268:Em-Doc:Para).

Within their narratives, participants often describe health professionals, particularly in acute care contexts, who they consider had acted inappropriately ( *“pinched”, “punched”, “stabbed”, “rubbed”, “hit”, “dropped”*), causing them injury:

*“A nurse came over and was openly accusing me of 'faking' the seizure, "Who the hell fakes a seizure?!". She then started doing things to try to get some sort of reaction from me (I was unresponsive at the time). I was on my front (where I'd ended up post-convulsions) […] She lifted the back of the bed, and when she got no reaction (because I couldn't react), she lifted it further, straining my stomach muscles to a very painful extent, and causing crushing back pain that continues to this day (over 5 months on). She then bent a pillow around my face, again to try and get a response, and this was very traumatic for me”* (ID40933009:Em-doc:Em-other).
*“My friends had called the ambulance […] the paramedic that came was so mean to me, I was very lethargic and out of it he rubbed my chest and left huge bruises and pinched me on my arm leaving my arm with huge bruises as well […] brought me in to the ER told the ER doc oh she is a faker. I had never had seizures before and never been diagnosed at this point. I kept having more, the nurse said she is having more the doc yells up she is faking leave her alone don't worry about her, when he came to see me he said neurology is too busy with real cases to be bothered to see me, a faker”* (ID43737533:Em-doc:Para).
*“The nurse […] put me in a wheelchair with force and started shouting at me and pushing my shoulder and head back into the chair. I was very woozy and didn't understand what was happening. My body started to shake, my eyes were open so I was clearly awake, the nurse went to do the sternum rub and instead punched my collar bone and started to rub her knuckles hard on that, she then pushed the wheelchair back into the wall and my head hit. She threatened to call the mental health down and said that 'she couldn't watch me all night have a fit' and that I was taking up everyone’s time and I was wasting the NHS resources and money”* (ID40931631:EM-Other).

### Consequences

Spontaneous descriptions regarding the adverse consequences of negative healthcare interactions frequently featured in participants’ responses.

Negative interactions, participants explain, can make them feel “ *frustrated*”, “ *afraid*”, “ *very upset*”, “ *humiliated*” and “ *ashamed*”:

*“Such hostility […] I always feel guilty, ghastly, 'failing to get better', etc. I had a (minor) head injury, just glued. I felt so humiliated by her antagonism when I was already emotionally really vulnerable”* (ID41134293:Staff-OHS).
*“One neurologist treated me like I wasn’t important because I didn't really have epilepsy. He just wanted to push me onto another neurologist to get me out of his office. He didn't really seemed to care about my feelings or what I had to say. I felt very ashamed walking out of his office, because I wasn't a real epilepsy patient”* (ID41447164:Gen-Neuro).
*“Several believe that it is just faked and that I have caused it […] being told you are wasting time and being made to feel guilty is very upsetting”* (ID40929177:All-HP).

At worst, they can leave them feeling *“horrified”*, *“hopeless”* or *“traumatised”*; and for some, had even *“resulted in suicide attempts”*:

*“… left feeling hopeless and untrustworthy of the health care profession. I went to where I went a period of 4 years without seeking medical treatment until I started having chronic pain issues”* (ID43737972:All-HP).
*“… I left feeling as if there was no hope and that I was no more than a joke to these nurses and physicians”* (ID41489268:EM-Doc:EM-other).
*“The majority of health professionals refer to the seizures as pseudo, and they translate this as fake. I already feel like a failure due to my inability to control the seizures, these experiences just go on to reinforce these feelings, and have resulted in suicide attempts”* (ID41610875:All-HP).

Participants often explain that the affect of negative medical interactions has left them with a *“dislike”*, *“difficulty trusting”* or *“fear”* of health professionals:

*“I really don't like going to the ER with seizures of any kind because I am afraid of them”* (ID43955993:Em-Doc:Em-Other).
*“It was an awful experience and why I have a dislike for paramedics and most of the medical profession”* (ID43728373:Para).
*““I felt deeply misunderstood and offended and it has affected me hugely […] I now have difficulty trusting healthcare professionals […] I fear hospitals, almost to a phobic extent […] It has affected me massively […] when you don't trust that you'll be treated appropriately by others when completely unable to explain or defend yourself, it's terrifying […] I don't think health professionals realise the potential consequences of their actions”* (ID40933009:Em-doc:Em-other).

Several describe that they now avoid disclosing or discussing NES with health professionals or seeking help for seizures; and some explain how they eschew medical help altogether:

*“I have been discouraged from even mentioning this issue with most doctors and nurses that I deal with, being told that if it's not epileptic it's not a "real" seizure and should not be even brought up ever. And yet, when I find myself on the floor, it sure feels real to me! This is not in any way something that I would want to invent, fake or choose to have if there were an option”* (ID40967712:All-HP).
*“Because of their attitudes and way I was treated I will never see anyone about any seizure activity”* (ID43349947:All-HP).
*“This treatment by the medical profession is just not good enough. I personally never want to have to see anyone on the medical profession ever again as I fear that any condition I may have will be attributed to NEAD”* (ID41438757:All-HP).

## Discussion

In line with previous research ^4, 19– 21^, our study participants experience negative medical interactions in several ways. The overarching narrative depicts a healthcare journey punctuated by poor, sometimes unethical and often detrimental medical encounters, which represent a fundamental breakdown in patient-provider relationships. Participants describe encountering health professionals who meet them with disbelief, suspicion, blame and judgment, and treat them with disdain and disrespect.

As reflected in participants’ responses, lack of practitioner knowledge and awareness of the condition is a decisive aspect. People with NES have previously reported limited understanding of the condition among medical professionals ^18^, who they explain are uncertain how to work with them ^17, 18^.

Research exploring health professionals’ views supports these assessments. Three-quarters of neuroscience nurses questioned reported limited knowledge of the illness ^14^. Surveys of neurologists, psychiatrists and emergency medicine professionals show all profess a greater understanding and knowledge of epilepsy than NES ^27– 29^, and family, internal, and emergency medicine practitioners express poor overall confidence in dealing with NES patients ^16, 30^.

Yet, according to our data, the negative experiences of study participants emerge from more than practitioners’ lack of awareness of NES and access to information about the condition - to the extent that it is available. In examining the challenges people with NES encounter when interacting with health professionals, their main experiences centre on blame and humiliation. In order to gain further understanding of their experiences - how they originate, what wider implications they have, and how they can be improved – it is useful to frame their experiences in relation to the phenomenon of stigma.

### Stigma

Stigma is associated with personal characteristics labelled as a mark of disgrace, difference and deviance. The historical origin of the term derives from the Greek ‘to puncture or brand’, and was later used to designated a mark of slavery or criminality; a branded Roman slave was known as a ‘stigmalic’ and the brand itself referred to as the ‘stigma’ ^31^.

Sociological conceptualisations of stigma can be traced to the symbolic interactionist perspective, with its basis in human interaction and social constructionism. Spanning the spectrum of physical and mental, overt and hidden, voluntary and involuntary, Goffman defines stigma as an *“*attribute that is deeply discrediting” ^32^. Those who are stigmatized deviate from cultural norms and values about how people ought to think, talk and act; it is contextual and relational rather than prescriptively defined ^33^. Actors within a sociological framework “create deviance by making the rules whose infraction constitutes deviance *”* ^34^. Applying these ‘rules’ to deviant individuals or groups means labelling them as *“*outsiders *”* ^34^; which can have profound affects on their self and social identity ^34, 35^.

Based on the visibility and nature of their “spoiled identities”, “discredited” individuals or groups are “disqualified from full social acceptance” and excluded from normal social interaction ^32^. The process is culturally moderated and reinforced. The method of induction to and practice of normative values, beliefs and behaviours is via the process of socialization ^36^. Group socialization and peer group pressure processes are perpetual self-reinforcing circuits of control, defining normality and reactions to deviations from it ^37^.

Tying these factors together, stigma has been conceptualised as entirely contingent on power imbalances in situations that allow the components of stigma (identification of differentness, the separation of labelled persons into distinct categories, the construction of stereotypes, status loss, and the execution of disapproval, rejection, exclusion, and discrimination) to unfold ^38^. It is within this framework that we interpret our participants’ experiences. Despite becoming less autocratic, doctor-patient interactions are still asymmetric, and the power to label and define medical situations primarily rests with health professionals ^39^.

***Identification of differentness and labelling.*** One of the most obvious ways our participants’ express they are defined in medical encounters is by being distinguished as not having epilepsy: “ *I wasn’t a real epilepsy patient*”. As their physical symptoms cannot be rationally explained by medical science, people with NES are designated as having a non-organic condition; which our participants describe is often demarcated by health professionals who explicitly refer to a lack of positive biomarkers, *“no abnormalities in the brain”*. The seizures are considered caused by psychological factors, and patients are labelled as having a psychogenic illness.

***Construction of stereotypes.*** Having received the psychogenic label, our participants describe being negatively stereotyped as “ *crazy*”, “ *lazy*”, “ *time-wasters*” and “ *fakers*”, and their character is defamed. These negative stereotypes are based on dominant cultural beliefs linking people with psychogenic illness to undesirable characteristics.

Self-control, restraint and regulation are highly valued character traits in our culture. Psychological understandings of NES defy these ideals and violate one of the main hallmarks of modernity in Western societies and medical systems: the individualistic “triumph of the will” ^40^; which emphasizes personal choice, followed by responsibility and accountability ^41^. Because the condition is seen as ‘all in their head’, participants recount being held accountable for onset of the illness (“ *like … I had somehow over-stressed myself to the point that I couldn’t just handle it*”) and liable for their symptoms. In the absence of empirical (biomedical) evidence, they experience accusations of faking seizures and are considered morally culpable; and relate explanations proposed for their intent: to fulfil some psychological need (“ *faking for attention*”) or malingering for secondary gain - most commonly, “ *to get out of work*”.

In line with our findings, previous studies describe people with NES who perceive health professionals to think them a “fraud” and suspect them of “staging” or “faking” their seizures ^17– 19, 42^, with inferred accusations of attention seeking ^18, 19^. Participants’ perceptions and experiences are reflected in research exploring the perspectives of health professionals. Up to half of neuroscience nurses questioned consider patients with conversion disorder manipulative ^14, 15^. Interviews with general neurologists show most view feigning and malingering as entangled with conversion disorder (where NES is grouped) ^27^, and survey research supports this assessment ^43^. Substantial proportions (up to 48%) of health practitioners in other clinical settings are also found to consider the seizures “voluntary” or “faked” ^15, 16^, with emergency medicine physicians significantly more likely to believe events are voluntarily induced than those in other specialties ^16^.

***Loss of status.*** According to our data, people labelled as having psychogenic illnesses lose the status of legitimate patient; participants describe being more-or-less treated as though they have “ *no medical problem*”. Their position as medical ‘outsiders’ appears reinforced by systems that struggle to place psychogenic conditions; which fall into neither camp on which our medical knowledge, curricula, or health services are built. Because it is not empirically verifiable, participants’ experiential realities – and even the ontological existence of their illness are challenged. Previous research shows that people with NES report that their symptoms are met with disbelief, the condition is not taken seriously, and the legitimacy of the illness is sometimes questioned by professionals ^17, 42, 44– 46^. However, ours is the first to describe those with the condition encountering health professionals who they report do not believe in the existence of the illness itself: “ *told that my condition didn't really exist*”. Practitioner denial of other medically unexplained physical conditions has previously been reported ^47^.

Because they are medically illegitimated, held morally culpable and stereotyped, people with NES do not attain the status of credible persons; and participants describe their narratives are considered suspect and discounted. Marginalisation of NES patient experiences, perspectives, and knowledge is a common complaint of people with NES in the literature ^17, 42, 44– 46^. In our data, disregard of NES patients experiential perspectives contributes to an ‘empathy gap’, characterised by professionals who (in the words of one participant) fail to recognise “ *the human cost of this illness*”. Practitioner empathy and compassion are considered altruistic values of modern medicine, but neither can be achieved without a “willingness to subject one’s mind to the patient’s world” ^48^; and to understand and believe their situation.

For people with NES, their eroded legitimacy and credibility seeps beyond the confines of the condition. In those whose illness is defined as psychogenic, minimal further medical investigation is recommended ^49^; and practitioners may be unwilling to investigate other ailments they present with, defining them as psychosomatic (“ *blamed my ear and sinus pain on my condition*”).

***Execution of disrespect.*** As participants experience a lack medical, moral and credible status, they are left open to being treated with disrespect. Previous studies have commented on patients with NES who perceive health professionals to display rude behaviours ^18, 19^; yet, these are rarely explicitly reported on in the literature. Nevertheless, a recent opinion piece suggests pejorative medical humour about the condition – and of people with NES, to be common in medical circles ^21^. The same authors report unpublished data from a survey of 120 patients with NES who describe frequent and explicit expressions of disbelief and contempt made to them or their family members by healthcare providers ^21^.

In our data, this contempt does not always end at poor interpersonal (communicative) treatment. Perhaps the most defining (and certainly the most shocking) feature of our participant narratives is the maltreatment they report receiving, particularly in acute care situations. In what often appears to be an effort to prove them as “ *fake*” – and unveil their moral character, some recount practitioner conduct that defies all ethical and professional obligations and standards; and which has not been previously reported in the literature to our knowledge. This practitioner conduct goes beyond the legitimate use of stimuli such as a sternum rub or pin prick test to determine if seizure activity is in status epilepticus: “bent a pillow around my face”, “shoved”, “head hit”.

### Consequences

For our participants, negative healthcare interactions can have wide-ranging consequences: emotionally, physically, and for their future healthcare. In line with our findings, previous studies show that negative interactions can leave those with the condition feeling dismissed, distressed, angry, frustrated and without hope ^17, 19, 44– 46^. Yet, none have previously reported the feelings of trauma and suicidal ideation directly associated with negative healthcare encounters expressed by some of our participants.

Previous studies have also found that negative medical encounters can discourage people with NES from seeking help ^17, 19, 44^, and is seen as a contributing factor to poor engagement and adherence with treatment ^20, 21, 45, 50^. However, ours is the first to report that some with the condition now avoid medical services altogether – probably because all other studies have recruited patients from clinical (predominately specialist) settings.

### Strengths and limitations

A major strength of this study is that data were collected anonymously - without researcher interference - and unconnected with any clinical setting. This approach allowed participants to describe their experiences without fearing consequences from healthcare providers. However, our approach means that we cannot say whether participants met minimum study criteria or have objectively verifiable NES diagnosed in accordance with International League Against Epilepsy Non-epileptic Seizures Task Force guidelines ^51^. We must also consider that the diagnosis of NES is notoriously complex and difficult, and some participants might be misdiagnosed. Video-EEG is the best-practice (‘gold standard’) diagnostic method ^52^; however, it is expensive and resource limited ^53^ and may not be feasible because of the low frequency of seizures ^54^.

Knowing that people with NES often experience difficult and challenging relationships with health professionals, we explicitly explored the facets of negative medical interactions. This does not necessarily mean our participants do not have positive experiences; indeed, additional data collected about their “single best interaction” with health professionals about NES, suggests many do. We incorporate this data in an upcoming paper ^55^ that seeks to identify what patients expect from health professionals, how their expectations are met, and how their expectations and experiences are socially constructed and structurally conditioned. However, we thought it more important to firstly explore and work to understand the negative ones, if those aspects are to be improved.

In some respects, the negative experiences our participants describe are similar to those involving patients with other medically unexplained physical symptoms (MUPS), for example, in terms of patient opposition to the psychologicalisation of their ailments and doctors who are suspicious patients derive secondary benefit from their symptoms (e.g. in relation to work or state benefits) ^56, 57^. Yet, important differences are also apparent. A main finding of our research is that many participants report experiencing negative interactions in acute care contexts. Compared to many other MUPS, NES are more likely to require emergency care; a significant proportion of seizure complaints seen in emergency departments are NES ^58^. The lack of a comparison group is a limitation of this study, and it is not known if patients with other contested illnesses would respond differently when asked to describe their worst health care encounter. A control group could add a great deal to the interpretation of our participants’ responses. For example, it might be the case that our participants are more frequently explicitly accused of “faking” their symptoms compared to other people with MUPS because NES are more directly observable from the outside; and there is a clear comparable organic disease, epilepsy. Differences between the health care experiences of different MUPS-patient groups, and those with comparable organic illness, could be usefully addressed in future studies.

Perhaps the greatest concern with the collection of internet-based patient information is the reliability and validity of the data obtained – and this should be considered as a limitation when interpreting the findings of this study. Yet, recent reviews suggest health data can be collected with equal or even better reliability in Web-based questionnaires compared to traditional approaches ^59– 61^. A web-based approach offers the added benefit of time for reflection and the ability to consider and correct information, which has been shown to improve data quality ^49^. There are also strong indications that web-based questionnaires are less prone to social desirability bias ^59, 62^.

Although our participants are geographically distributed across Western nations, their stories are remarkably similar. Nevertheless, our population was self-selected as people with NES who have access to the Internet and are members of online support groups, and there might be important differences between our participants and others with the condition. A likely bias it that those who chose to participate in our study have experienced more, or more severe, negative medical interactions than those who did not. In addition, men are underrepresented in our sample. Our data is probably also biased toward higher functioning patients, as those more seriously affected might have been unable to participate. Due to the way the research was designed, there is no way to assess survey response rate. Using the number of surveys started (289) as a proxy denominator suggests a completion rate of 49%.

As with all methods that seek to understand and describe people’s personal experiences (the emic or insider’s viewpoint), we recognise that our findings are more easily influenced by personal biases than some other methods and approaches. Nevertheless, qualitative approaches are particularly responsive to exploring patient experiences - and we consider our methods appropriate to generate insights into the healthcare experiences of those with NES, and to offer possible explanations as to how and why NES patients experience the quality of healthcare services they do.

## Conclusion

The quality of health care interactions for people with NES requires urgent improvements. Our findings show that in addition to medical knowledge, to the extent that it is available, practitioners need to be conscious of making and acting on adverse moral appraisals when interacting with this patient group. Normative value judgements arising from psychogenic understandings of NES are stigmatising and restrict professional displays of respectful patient-centred care. Psychological factors are culturally interpreted as negative character traits. Just as people with epilepsy were once blamed and shamed, those whose seizures are labelled psychogenic are negatively stereotyped and held morally culpable. Whether viewed as a sign of weakness ^63^ or as simple malingering ^64^, somatic symptoms without objectively knowable and observable organic pathology become seen as somehow self-inflicted ^64, 65^.

In the absence of technologically identifiable causative mechanism(s) that externally validate the reality of their illness and apportion liability (to the soma, as opposed to - or as well as - the psyche), it is difficult to see how people with NES can truly acquire the status of legitimate and credible patients within the modern biomedical paradigm. This situation calls for health professionals who recognise patient experiences, reflect on the moral judgements they convey, and thoughtfully consider the potential consequences of their actions in relation to the fundamental principle of *primum non nocere*. Those with NES ask practitioners to “ *believe the experiences I tell you*” and accept that “ *there are just some things that the brain does that we just don’t have definite answers for*”. This should not be too much to ask.

## Ethical statement

Ethical clearance was given by Nelson Mandela University Human Research Ethics Committee (NMU-HREC) on 22nd April 2016 (REF:H16-RTI-RCD-002), where the second author was a visiting researcher at the time of data collection. The survey was hosted by a UK (and EU) data-protection compliant provider. Potential participants were guided through an online study protocol detailing the purpose of the study, and data protection, ethical compliance and complaint procedures. All participants gave informed electronic consent to participate, and did so again to confirm survey completion and authorise submission of their data for use in the research project.

## Data availability

The data referenced by this article are under copyright with the following copyright statement: Copyright: © 2017 Robson C and Lian OS

Data is not open-source, and access to anonymised data is restricted in line with Nelson Mandela University Human Research Ethics Committee (NMU-HREC) approval. NMU-HREC approval provides concession for re-use of anonymised data, which participants agreed to when giving informed consent. Access to anonymised data supporting this publication can be granted to 'bona fide' researchers. To expedite the process, an amendment was granted by the NMU-HREC for interested parties to apply for a Chair’s Action to access data in order to verify results underpinning publications (NMU-HREC, 20 ^th^ June 2017, REF:H16-RTI-RCD-002/Amendment). A pro forma cover letter and application form is attached as Supplementary information, and provides a detailed overview of the procedure and conditions and terms of access. Researchers are also able to use this process to apply for access to the data for secondary research, where the use of the data is to study a problem that was not the focus of the publication. It is at the Chairperson’s discretion as to whether a full Research Ethics Review is required, and applicants will be advised accordingly. Anonymised data will only be released to named parties once approval for re-use is granted by NMU-HREC Chair’s Action or Committee approval and an ethical clearance letter provided. The Chairperson or NMU-HREC may request access to the data before it is released, to ensure it is truly anonymised.

## Supplementary material

**Supplementary File 1: Application for NMU-HREC Chair’s Action.**

The pro forma cover letter and application form can be completed by ‘bona fide’ researchers and submitted to the Chairperson of the NMU-HREC in order to gain access to anonymised data. The document details current NMU-HREC approvals and the procedure and conditions and terms of access to anonymised data underpinning this publication. Interested parties are asked to provide their particulars (and those of co-applicants, if applicable), to describe the proposed use of anonymised data, and to declare they and any co-applicants will abide by the terms and conditions of data use. Researchers are also able to use this process to apply for access to the data for secondary research, where the use of the data is to study a problem that was not the focus of the publication. It is at the Chairperson’s discretion as to whether a full Research Ethics Review is required, and applicants will be advised accordingly.
