# Supplementary material for: “ Blaming, shaming, humiliation”: Stigmatising medical interactions among people with non-epileptic seizures
Source: Wellcome Open Res. 2017 Oct 24;2:55. Originally published 2017 Jul 24. [Version 2] doi: 10.12688/wellcomeopenres.12133.2 (PMC5664997; doi:10.12688/wellcomeopenres.12133.2)
Supplement: Supplementary file 1 [file wellcomeopenres-2-14021-s0000.tgz › 2b0fc3b5-cb48-46c7-93de-edbd895229b5.docx]

**APPLICATION FOR CHAIRPERSON’S ACTION**

**NMMU RESEARCH ETHICS COMMITTEE (HUMAN)**

Request for access to anonymised data

REF:H16-RTI-RCD-002

Date: [INSERT]

**For attention of the Chairperson of NMMU Research Ethics Committee (Human)**

Dear Chairperson,

Please find attached a request for access to anonymised data pertaining to the study, ‘Listening to people with psychogenic non-epileptic seizures’ (REC-Human: H16-RTI-RCD-002), as detailed in section 1.

I understand that access to the anonymised data will only be granted to 'bona fide' researchers. Following [MRC guidelines](http://www.mrc.ac.uk/documents/pdf/data-sharing-from-population-and-patient-studies/), a bona fide researcher is a person with 1) the professional expertise and experience to conduct bona fide research and 2) a formal relationship with a bona fide research organisation that requires compliance with appropriate research governance and management systems. A bona fide research organisation is one that has the capability to lead or participate in high quality, ethical research. It will have a public commitment to adhere to recognised research and information governance good practice. I include my particulars and that of co-applicants (if applicable) in section 2. I understand that the Chairperson (or designated others) might conduct checks to confirm my standing, and that of co-applicants (if applicable).

Access is requested to anonymised data resulting from study questions detailed in section 3.

Access to the anonymised data is requested [delete A or B as appropriate]

[A] to verify results underpinning the publication, [INSERT PUBLICATION CITATION].

[B] for secondary research, where the use of the data is to study a problem that was not the focus of the publication, [INSERT PUBLICATION CITATION]. Information relating to the planned secondary research is detailed in section 4 of the document.

I understand that it is at the Chairperson’s discretion as to whether a full Research Ethics Review is required.

Declarations pertaining to the use of the data and the conduct of applicants are provided in section 5. Details of persons who this application has been submitted/copied to are provided in section 6.

I understand that the data requested can be only be released by the data-manager (Dr Catherine Robson) to named parties once approval for re-use is granted by Chairperson’s Action or Research Ethics Committee approval, and a clearance document/certificate is provided. I understand that the Chairperson or Research Ethics Committee may request access to the data before it is released, to ensure it is truly anonymised.

Yours sincerely,

[INSERT NAME OF PRIMARY APPLICANT]

| **1. Details of NMU Research Ethics Committee (Human) Approval** | |
| --- | --- |
| **Institution:** | Nelson Mandela University |
| **Research Ethics Committee:** | NMU RESEARCH ETHICS COMMITTEE (HUMAN) |
| **Title of study:** | LISTENING TO PEOPLE WITH PSYCHOGENIC NON-EPILEPTIC SEIZURES |
| **Ethics clearance reference number:** | H16-RTI-RCD-002 (and H16-RTI-RCD-002/Amendment) |
| **Clearance certificate dated:** | 22 APRIL 2016 (20 JUNE 2017/Amendment) |
| **Primary Responsible Person (PRP)** | Professor B Pretorious  Research Capacity Development, Office 1314, Summerstrand Campus (South)  [blanche.pretorius@nmmu.ac.za](mailto:blanche.pretorius@nmmu.ac.za) |
| **Principal Investigator (PI) and Data-Manager** | Dr C Robson  Research Capacity Development, Office 1314, Summerstrand Campus (South)  [catherine.robson@nmmu.ac.za](mailto:catherine.robson@nmmu.ac.za) and [catherinemaryrobson@gmail.com](mailto:catherinemaryrobson@gmail.com) |

| **2a. PARTICULARS OF PRIMARY APPLICANT** | |
| --- | --- |
| **Name:** |  |
| **Designation:** |  |
| **Institutional affiliation:** |  |
| **Full postal address:** |  |
| **Contact telephone number:** |  |
| **Email address:** |  |

| **2b. PARTICULARS OF CO-APPLICANT (if applicable)** | |
| --- | --- |
| **Name:** |  |
| **Designation:** |  |
| **Institutional affiliation:** |  |
| **Full postal address:** |  |
| **Contact telephone number:** |  |
| **Email address:** |  |

| **2c. PARTICULARS OF CO-APPLICANT (if applicable)** | |
| --- | --- |
| **Name:** |  |
| **Designation:** |  |
| **Institutional affiliation:** |  |
| **Full postal address:** |  |
| **Contact telephone number:** |  |
| **Email address:** |  |

| **2d. PARTICULARS OF OTHER CO-APPLICANTS (if applicable)** |
| --- |
| Please list additional co-applicants here, including their name, designation, institutional affiliation, full postal address, contact telephone number and email address. |

| **3. Request for access to anonymised data in relation to study questions:**  [For Chairperson or REC: Indexing corresponds to the survey questionnaire submitted as part of REC approval, appendix 1B] |
| --- |
| SOCIO-DEMO1: Country of residence |
| SOCIO-DEMO3: Age |
| SOCIO-DEMO4: Gender |
| SOCIO-DEMO5: Relationship status |
| SOCIO-DEMO7: Employment status |
| SEIZURES-DIAG1: NES diagnostic status |
| SEIZURES-DIAG2: Diagnosis of NES received from [type(s) of health professional] |
| SEIZURES-DIAG4-5-6: Prior erroneous diagnosis of epilepsy |
| SEIZURE-HIST1: Time from onset of NES |
| INT-HEALTH-PROFS4: Single worst interaction [type(s) of HP] |
| INT-HEALTH-PROFS5: ‘Single worst’ interaction [description-open] |
| INT-HEALTH-PROFS7: ‘Anything else’: Interacting with HP [description-open] |

| **4. Proposed use of anonymised data** | |
| --- | --- |
| Access to anonymised data resulting from study questions detailed in section 3 is requested to verify results underpinning the publication, [INSERT PUBLICATION CITATION]. | [Please indicate YES or NO] |
| Access to anonymised data resulting from study questions detailed in section 3 is requested for secondary research, where the use of the research data is to study a problem that was not the focus of the publication, [INSERT PUBLICATION CITATION]. | [Please indicate YES or NO] |
| If you have requested access to the anonymised data for secondary research purposes, please briefly describe (using no more than 1 page of A4) the proposed study using the headers detailed below. It is at the Chairperson’s discretion as to whether a full Research Ethics Review is required. The Chairperson may contact you for further information.  Proposed study title  Aims and objectives of the study  Rationale for the study  Methods to be used |  |

| **5. Declarations**  **If access to the anonymised data (as detailed in section 3) is granted by Chairperson’s Action, applicants agree to:** | | | | |
| --- | --- | --- | --- | --- |
| Undertake to abide by the ethical principals underlying the Declaration of Helsinki [(available here)](http://www.who.int/bulletin/archives/79(4)373.pdf) and to abide by the Nelson Mandela University’s Research Code of Conduct [(available here)](http://ebeit.nmmu.ac.za/ebeit/media/Store/documents/Research%20Guidelines/PlanningTheResearch/CODE-OF-CONDUCT-FOR-RESEARCHERS.pdf). | | | | |
| Undertake to adhere to the proposed use of anonymised data (as detailed in Section 4) without agreed deviation. | | | | |
| Undertake to inform the Chairperson or Research Ethics Committee of any changes in protocol that would have ethical implications for the research. | | | | |
| Securely store the data, and not allow any other persons - with the exception of those listed in this application and for whom approval for use is granted - access. | | | | |
| Acknowledge Wellcome Trust’s support in any publication directly relating to the work undertaken as a result of the data received, referencing application number: 200923/Z/16/Z. | | | | |
| **I hereby confirm that I have read, understood and will comply with the above arrangements or procedures.** | | | | |
| **Applicant name** | **Designation** | **Institutional affiliation** | **Signature** | **Date** |
|  |  |  |  |  |
|  |  |  |  |  |
|  |  |  |  |  |

| **6. Application submitted /copied to:**  **The application has been submitted/copied to the following persons:** | | | |
| --- | --- | --- | --- |
| **Name** | **Designation** | **Email Address** | **Confirmation (check)** |
| Prof. C Cilliers | Chairperson of the Nelson Mandela University Research Ethics Committee (Human) | [charmain.cilliers@nmmu.ac.za](mailto:charmain.cilliers@nmmu.ac.za) |  |
| Prof. B Pretorious | Primary Responsible Person (Research Capacity Development) | [blanche.pretorius@nmmu.ac.za](mailto:blanche.pretorius@nmmu.ac.za) |  |
| Dr Catherine Robson | Principal Investigator and Data-Manager | [catherine.robson@nmmu.ac.za](mailto:catherine.robson@nmmu.ac.za) and [catherinemaryrobson@gmail.com](mailto:catherinemaryrobson@gmail.com) |  |
| If you encounter any problems you can contact the following persons, who may be able to assist you: | | | |
| Ms Ursula Spies | Committee Officer – Governance Administration | [Ursula.Spies@nmmu.ac.za](mailto:Ursula.Spies@nmmu.ac.za) | +27 41 504 3140 |
| Mr Imtiaz Khan | Administrative Management Officer – Research Capacity Development | [Imtiaz.Khan@nmmu.ac.za](mailto:Imtiaz.Khan@nmmu.ac.za) | +27 41 504 4329 |
| Contact details for (current) REC Chairperson’s, Secretariats, and Administrative Management Officers are [available here](http://rcd.nmmu.ac.za/Research-Ethics/Research-Ethics-Committee-Human-(REC-H). | | | |

| **7. Further information** |
| --- |
| Should the Chairperson advise that a **full Research Ethics Committee review** is needed, please consult the following procedures and documents [available here](http://rcd.nmmu.ac.za/Research-Ethics/Research-Ethics-Committee-Human-(REC-H). Particularly:   - Procedures for external parties wishing to conduct research at NMU; - Details about the application process; - REC application form; and - Important dates for your diary.   After reading the information available above, if you are need of assistance please contact the Administrative Management Officer, Mr Imtiaz Khan, whose details are provided above (section 6). |
