# Supplementary material for: “ Blaming, shaming, humiliation”: Stigmatising medical interactions among people with non-epileptic seizures
Source: Wellcome Open Res. 2017 Oct 24;2:55. Originally published 2017 Jul 24. [Version 2] doi: 10.12688/wellcomeopenres.12133.2 (PMC5664997; doi:10.12688/wellcomeopenres.12133.2)
Supplement: Peer review history

# Peer review: Referee response for version 2

**DOI:** 10.21956/wellcomeopenres.14021.r27277

**Published:** 2017-11-01

**Authors:** Benjamin Tolchin

**Affiliations:** 1 Department of Neurology, Yale University School of Medicine, New Haven, CT, USA

Thank you for your thoughtful responses to the reviewers' comments. I feel that my initial comments are sufficiently addressed.

I have read this submission. I believe that I have an appropriate level of expertise to confirm that it is of an acceptable scientific standard.

# Peer review: Referee response for version 2

**DOI:** 10.21956/wellcomeopenres.14021.r27276

**Published:** 2017-10-27

**Authors:** Christopher Dowrick

**Affiliations:** 1 Institute of Psychology Health and Society, University of Liverpool, Liverpool, UK

Thank you for your thoughtful and comprehensive responses to my comments.

I have read this submission. I believe that I have an appropriate level of expertise to confirm that it is of an acceptable scientific standard.

# Peer review: Referee response for version 1

**DOI:** 10.21956/wellcomeopenres.13132.r24460

**Published:** 2017-10-02

**Authors:** Pia H. Bülow

**Affiliations:** 1 School of Health and Welfare, Jönköping University, Jönköping, Sweden

This qualitative study of how people with non-epileptic seizures (NES) describe their experiences of their “single worst interaction with a health professional about NES” is interesting and important since it illuminates the difficulties people experience when suffering from something that does not fit into the biomedical norms of what could be regarded as a “proper” disease.

 Methodologically, I find the formulation of the requests for the free-text responses which are analysed, very interesting. Starting with asking the participants to “think about the single worst interaction you have had with a health professional about NES”, followed by the request to point out which category of health professional involved in the interaction in mind, and then another request to describe what happened during this encounter (“what you disliked about the interaction”) seems to me as a fruitful way to elicit memories and stories of a certain kind, that is here about really bad encounters with health professionals. This arrangement resembles William Labov’s classical study of narratives from the inner city (Labov, 1972) ^1^.

 However, I find the one-sided focus on negative experiences problematic especially since the focus is “the single worst interaction”. I do understand the authors wish to give the negative aspect space. But even if this is the most difficult part for people with NES, and what calls for improvement in the health care, a two-sided focus in the same article could offer a more nuanced picture.

 Another methodological problem I see, is that participants were recruited from online support groups only. This arrangement is practical for reaching people with a certain illness/diagnosis but also implies risks for biases. The authors discuss the limitation of their choice noting that people with NES who does not have access to Internet will be excluded. However, not everyone with a certain illness would like to join a support group. Additionally, although the presented analysis draws on written accounts and narratives (my interpretation) given as free-text responses, and even though the authors claim to analyse these “from a social-constructionist perspective”, they do not discuss other possible biases related to support groups as a kind of sub group of people with NES. Other studies using data from support groups (and similar groups) for other problems than NES have shown collective narrating and co-narrating as important aspects of the interaction in support groups (Bülow, 2004; Cain, 1991; Steffen, 1997) ^2^^-^^4^. Mutual sharing of information as well as mutual sharing of experiences in support groups could be reflected in the free-text responses. This as well as the possibility for more engaged and informed respondents might be interesting to discuss.

 The result, presented under six themes, offers a richness of citations from the collected data. In this part the character of data as accounts and narratives are pointed out using verbs such as the participants “describe”, “explain”, “report” etc. In the discussion, however there is sometimes a tendency of changing the narrated experiences into something else like “people with NES are held accountable for onset of the illness”.

 The use of the concept of stigma in the discussion is relevant and intelligible. Still, there are some gaps in the line of reasoning when tying different factors together and when the authors move from dealing with the participants’ recounted experiences and the author’s further interpretation of these. One example is the description of how constructions of stereotypes like “lazy, crazy, time-wasters and fakers” become the obvious consequence of when seizures are explained as “caused by psychological factors” and “labelled as having a psychogenic illness”. There are many illnesses which are thought to be caused of psychological factors like for instance mental illnesses in general. However, mental illnesses are not stigmatizing in relation to health professionals but in society generally speaking. The stigmatizing in relation to health professionals could be something of certain interest for the study of how people with NES experience encounters in health care contexts. As a social scientist interested in narratives about less visible illnesses such as chronic fatigue syndrome, burnout and similar contested illnesses, I wonder about the character of NES with visible symptoms resembling epilepsy for which there exists biomedical explanations and which are lacking for NES, and how this influence the encounters with health professionals. One possibility to elaborate this aspect is to consider the medical history about diagnoses like hysteria and how NES is different compared to less visible contested illnesses. The visibility of NES is mentioned in the conclusion but could be elaborated on.

 Finally, as this study shows that the participants “main (negative) experiences centre on blame and humiliation” maybe the analysis more obviously should be grounded on the free-text responses as accounts explaining the challenges encounters with health professionals implies for people with NES.

I have read this submission. I believe that I have an appropriate level of expertise to confirm that it is of an acceptable scientific standard, however I have significant reservations, as outlined above.

## References

[1] LabovW: Language in the Inner City. *Philadelphia: University of Pennsylvania Press*.1972;

[2] BülowP: Sharing Experiences of Contested Illness by Storytelling. *Discourse & Society*.2004;15(1) : 10.1177/0957926504038943 33-53 10.1177/0957926504038943

[3] CainC: Personal Stories: Identity Acquisition and Self-Understanding in Alcoholics Anonymous. *Ethos*.1991;19(2) : 10.1525/eth.1991.19.2.02a00040 210-253 10.1525/eth.1991.19.2.02a00040

[4] SteffenV: Life stories and shared experience. *Soc Sci Med*.1997;45(1) :99-111 920327510.1016/s0277-9536(96)00319-x

# Response

**Published:** 2017-10-13

**Authors:** Catherine Robson

**Affiliations:** University of Tromsø, Norway

We would like to thank the reviewers for their insightful comments; which have added more depth, substance and clarity to this paper. We are especially pleased that reviewers from different disciplines and areas of practice (a GP, neurologist and social scientist) took the time to thoughtfully review our work – and we sincerely thank you. We address the comments in turn below, removing some background text that did not require action.

**Pia H. Bülow**, School of Health and Welfare, Jönköping University, Jönköping, Sweden  

 1) I find the one-sided focus on negative experiences problematic especially since the focus is “the single worst interaction”. I do understand the authors wish to give the negative aspect space. But even if this is the most difficult part for people with NES, and what calls for improvement in the health care, a two-sided focus in the same article could offer a more nuanced picture.

 This paper was borne out of a desire to address (anecdotally - as we, and the patients we talk to see it) one of the most troublesome aspects of living with medically unexplained seizures – poor quality healthcare encounters. To explore and draw attention to these facets we made a (very) conscious decision to only focus on negative encounters. As described in the introduction, there are many reports of people with NES who describe negative interactions as common and expected, but there is scant literature as to what they perceive unhelpful/ adverse about these encounters, and why these situations might occur. Hence, the reason for our focus. Notably, there is also a growing body of literature exploring the perspectives of health professionals, which suggests they find interactions with NES patients “tough”, “uncomfortable”, “challenging” and “frustrating”. Therefore, we thought the study focus apt.

 We agree a two-sided approach would offer a more nuanced picture of patients’ experiences. However, the volume of data (and our desire to explore this considerable depth and present a rich selection of quotes) and what we thought the most important priority (as described above) prevented this. We have, however, added a reference to a paper that incorporates other (“positive”) data: Lian, O.S. and C. Robson, Socially constructed and structurally conditioned conflicts in territories of medical uncertainty, *Social Theory & Health*, (under review).

 2) Another methodological problem I see, is that participants were recruited from online support groups only. This arrangement is practical for reaching people with a certain illness/diagnosis but also implies risks for biases. The authors discuss the limitation of their choice noting that people with NES who does not have access to Internet will be excluded. However, not everyone with a certain illness would like to join a support group. Additionally, although the presented analysis draws on written accounts and narratives (my interpretation) given as free-text responses, and even though the authors claim to analyse these “from a social-constructionist perspective”, they do not discuss other possible biases related to support groups as a kind of sub group of people with NES. Other studies using data from support groups (and similar groups) for other problems than NES have shown collective narrating and co-narrating as important aspects of the interaction in support groups (Bülow, 2004; Cain, 1991; Steffen, 1997) ^2^ ^-^ ^4^. Mutual sharing of information as well as mutual sharing of experiences in support groups could be reflected in the free-text responses. This as well as the possibility for more engaged and informed respondents might be interesting to discuss.

 This is an interesting consideration. However, the supporting literature mentioned relates to ethnographic observation of regular (e.g. weekly) “in-person” meetings (each exploring a particular setting/context). Our data is taken from a survey advertised to members of 20 support groups, and participants were widely geographically distributed (albeit possibly connected via online avenues). We agree that a degree of co-construction/collective narration is possible, but given the research design probably sloping towards the minimal. We put a lot of thought into identifying limitations (with more now added). At 800+ words in length, we have dedicated much more text to this section than usually allowable in an article. In terms of possible biases related to support groups we do say that “… our population was self-selected as people with NES who have access to the Internet and are members of online support groups, and there might be important differences between our participants and others with the condition. A likely bias it that those who chose to participate in our study have experienced more, or more severe, negative medical interactions than those who did not”.

 3) The result, presented under six themes, offers a richness of citations from the collected data. In this part the character of data as accounts and narratives are pointed out using verbs such as the participants “describe”, “explain”, “report” etc. In the discussion, however there is sometimes a tendency of changing the narrated experiences into something else like “people with NES are held accountable for onset of the illness”.

 Apologies, the wording in the discussion reflects analysing participant experiences according to the overarching narrative, but we agree this cannot be extrapolated to people with NES more generally. We have edited relevant text and included words such as “perceive”, “depict”, “relate”, “recount”, etc.

 4) The use of the concept of stigma in the discussion is relevant and intelligible. Still, there are some gaps in the line of reasoning when tying different factors together and when the authors move from dealing with the participants’ recounted experiences and the author’s further interpretation of these. One example is the description of how constructions of stereotypes like “lazy, crazy, time-wasters and fakers” become the obvious consequence of when seizures are explained as “caused by psychological factors” and “labelled as having a psychogenic illness”. There are many illnesses which are thought to be caused of psychological factors like for instance mental illnesses in general. However, mental illnesses are not stigmatizing in relation to health professionals but in society generally speaking. The stigmatizing in relation to health professionals could be something of certain interest for the study of how people with NES experience encounters in health care contexts.

 It is interesting you mention this, as in the early rounds of data analysis I often posited questions such as: “would a health professional accuse a person with schizophrenia of “faking” … express contempt for them, if not – why not”? The difference, we believe, comes back to the psychogenic element (physical symptom(s) thought caused by psychological factors). Mental symptoms caused by psychological factors seem easier to accept. Mental illness might also be less stigmatising in health care contexts as it is an established area of practice. People with psychogenic conditions tend to fall through the cracks (in the case of NES: neurology and psychiatry). In relation to psychogenic conditions we tried to cover these angles in the text (e.g. Discussion, Loss of status): treated as though they have “no medical [physical] problem”, “empirical variability”, and “ … reinforced by systems that struggle to place psychogenic conditions; which fall into neither camp on which our medical knowledge, curricula, or health services are built”. Another interesting point to consider is that psychological symptoms caused by physical factors also appear palatable (in both medical and social spheres). Unfortunately, a fuller analysis and discussion is beyond the bounds of this paper (and will undoubtedly stray into the philosophical: see for example Dekkers and van Domburg, 2000), but could be usefully incorporated in future work.

 As a social scientist interested in narratives about less visible illnesses such as chronic fatigue syndrome, burnout and similar contested illnesses, I wonder about the character of NES with visible symptoms resembling epilepsy for which there exists biomedical explanations and which are lacking for NES, and how this influence the encounters with health professionals One possibility to elaborate this aspect is to consider the medical history about diagnoses like hysteria and how NES is different compared to less visible contested illnesses. The visibility of NES is mentioned in the conclusion but could be elaborated on.

 This ties into a point made by Prof. Dowrick (2b) - We have reflected on the possible similarities/differences between the negative experiences of people with NES and those reported by people with other MUPS, and have dedicated a paragraph to this in the Strengths and limitations section. Please also refer to our text above. Regarding the medical history of NES (hysteria with convulsions, catalepsy, etc.) we draw on this in the paper now referenced: Lian, O.S. and C. Robson, Socially constructed and structurally conditioned conflicts in territories of medical uncertainty, *Social Theory & Health*, (under review).

 5) Finally, as this study shows that the participants “main (negative) experiences centre on blame and humiliation” maybe the analysis more obviously should be grounded on the free-text responses as accounts explaining the challenges encounters with health professionals implies for people with NES.

 Agree, and we have incorporated this in the abstract, the introduction and in methods.

# Peer review: Referee response for version 1

**DOI:** 10.21956/wellcomeopenres.13132.r26084

**Published:** 2017-09-28

**Authors:** Benjamin Tolchin

**Affiliations:** 1 Department of Neurology, Yale University School of Medicine, New Haven, CT, USA

This is a qualitative study exploring how people with non-epileptic seizures (NES, also known as psychogenic non-epileptic seizures [PNES] or non-epileptic attack disorder [NEAD]) experience interactions with healthcare professionals. It is an extremely valuable contribution to an existent but very limited literature describing the experience of living with NES. The authors utilize free-text responses to an anonymous web-based survey from 135 subjects who reported receiving a diagnosis of NES made by a health professional. Among 86 items on the survey, subjects were prompted to “think about the single worst interaction you have had with a health professional about NES” and to write about “what you disliked about the interaction.” Subjects’ responses to these prompts and to a more general question (“is there anything else you would like to tell us about your experiences”) formed the basis for the qualitative data. The authors then used a thematic discourse analysis approach to generate six key themes such as negative and disrespectful encounters, lack of knowledge and awareness, illegitimate patients, etc.

 The survey results and analysis are clinically relevant and important in that they give a detailed and very consistent picture of the substantial pain and humiliation experienced by persons with NES in their interactions with medical providers – especially neurologists and emergency medicine providers. The analysis also highlight specific behaviors and communication styles that contribute to patients’ suffering. As a practicing neurologist the quotations were frankly painful to read because they reflected the hurt inflicted by behaviors I have often witnessed or heard discussed and sometimes performed myself. Neurologists, emergency medicine physicians, and other healthcare providers and trainees can gain invaluable insight into their patients’ perspectives from this study and may concretely modify their actions and communication strategies as a result.

 Having said this, there are several methodological concerns that raise questions about the validity and interpretation of the data. I have marked particularly concerning issues with an asterisk.

 *1) The main inclusion criterion was having received a diagnosis of NES from a health professional. There was no requirement that subjects have a diagnosis of documented NES confirmed by video-EEG capture of typical events, with review by neurologists experienced in the diagnosis of epilepsy and NES, as per requirements published by the International League Against Epilepsy (ILAE) ^1^. Studies demonstrate that experienced neurologists make errors in discriminating epileptic seizures from NES based on visual inspection of the seizure alone without EEG confirmation, and other medical providers have substantially lower discrimination for NES, approaching chance in some cases ^2^. Eighty-five percent of subjects in the study reported a “formal (certain or highly likely) diagnosis of NES” but it is not clear that these subjects were diagnosed in accordance with ILAE standards.

 2) Subjects were recruited through advertisements to NES support groups to participate in an anonymous online survey. There was no verification that respondents actually met the limited inclusion criteria described above. The authors acknowledge this limitation. They argue “recent reviews suggest health data can be collected with equal or… better reliability in Web-based questionnaires compared to traditional approaches.” Citations should be given here. Additionally, the diagnosis of NES is notoriously complex and difficult, subject to confusion and error at many steps. As such there is a legitimate question as to how many subjects actually had NES.

 3) Similarly there could be no verification of subjects’ reported experiences. While some of the reported interactions are very plausible, others sound like the subjects may be interpreting or elaborating dismissive but less dramatically offensive behavior on the part of a provider (“he laughed in my face at the diagnosis of FND,” “I can remember her just standing over me… shouting ‘get up you are wasting my time, why do I have to put up with patients like you’”). There is great value in these reports because they illustrate the subjects’ perceptions and suffering, but it’s questionable whether they should be interpreted literally. The authors generally discuss the responses as illustrations of patients’ perceptions but on occasion appear to interpret reports literally (“participants recount practitioner conduct that defies all ethical and professional obligations and standards; and which has not been previously reported in the literature to our knowledge”).

 4) There is a (perhaps understandable) lack of attention to the motivations and subjective experiences of the providers involved in the reported interactions. Some of the most disturbing quotations describe emergency medicine (EM) physicians apparently physically torturing subjects, and then crowing that the subjects are “faking” when they respond to physical discomfort (“He stabbed my arms with a needle whilst I was paralyzed to prove I was faking…. He kept telling me I was faking”). The context behind such encounters is that EM physicians need to determine within 5 minutes whether a patient with ongoing seizure activity is in status epilepticus (a life-threatening condition which should be aggressively treated with potentially dangerous sedative medications, possibly leading to intubation and iatrogenic injury or death) or NES (which should not be treated with medications). Unlike neurologists in epilepsy monitoring units, EM physicians must usually make this determination without the aid of EEG monitoring. As a result they often resort to noxious stimuli (sternal rub, nailbed pressure) in an attempt to get an accurate measure of responsiveness. While stabbing a patient with a needle is almost certainly unnecessary (and a diagnosis of NES should not be equated with faking), such behavior is often motivated by concern for the patient’s life rather than a desire to trick the patient. It is invaluable for physicians to understand how patients perceive this behavior, but it also important for patients and researchers to understand the perceptions and motivations of healthcare providers. Increasing communication between patients and providers and between disciplines, as exemplified by this study, may aid in both goals.

 *5) The lack of a control group raises an important question: how different -- how much worse – were the experiences of the subjects in comparison to the experiences of patients with other chronic illnesses? Were researchers to prompt groups of patients with other chronic illnesses to think of their worst healthcare experiences, it seems likely that many would recount truly horrific experiences. A control group with a different chronic illness would add a great deal to the interpretation of the subjects’ responses, and failing that a direct comparison by the authors to similar web surveys of different patient groups would be helpful.

 *6) The respondents to the survey are very likely biased toward those with more negative experiences, whereas those without intense negative experiences would presumably have less motivation to complete a lengthy and uncompensated online survey. The authors appropriately identify this likely bias as a limitation.

 Overall, these concerns are largely addressable and the study provides very important insights into patients’ experience of NES and health provider behavior. I therefore approve this study with reservations.

I have read this submission. I believe that I have an appropriate level of expertise to confirm that it is of an acceptable scientific standard, however I have significant reservations, as outlined above.

## References

[1] LaFranceWCBakerGADuncanRGoldsteinLHReuberM: Minimum requirements for the diagnosis of psychogenic nonepileptic seizures: a staged approach: a report from the International League Against Epilepsy Nonepileptic Seizures Task Force. *Epilepsia*.2013;54(11) : 10.1111/epi.12356 2005-18 10.1111/epi.12356 24111933

[2] SeneviratneURajendranDBruscoMPhanTG: How good are we at diagnosing seizures based on semiology?. *Epilepsia*.2012;53(4) : 10.1111/j.1528-1167.2011.03382.x e63-6 10.1111/j.1528-1167.2011.03382.x 22242732

# Response

**Published:** 2017-10-13

**Authors:** Catherine Robson

**Affiliations:** University of Tromsø, Norway

We would like to thank the reviewers for their insightful comments; which have added more depth, substance and clarity to this paper. We are especially pleased that reviewers from different disciplines and areas of practice (a GP, neurologist and social scientist) took the time to thoughtfully review our work – and we sincerely thank you. We address the comments in turn below, removing some background text that did not require action.

**Benjamin Tolchin**, Department of Neurology, Yale University School of Medicine, New Haven, CT, USA  

 *1) The main inclusion criterion was having received a diagnosis of NES from a health professional. There was no requirement that subjects have a diagnosis of documented NES confirmed by video-EEG capture of typical events, with review by neurologists experienced in the diagnosis of epilepsy and NES, as per requirements published by the International League Against Epilepsy (ILAE) ^1^. Studies demonstrate that experienced neurologists make errors in discriminating epileptic seizures from NES based on visual inspection of the seizure alone without EEG confirmation, and other medical providers have substantially lower discrimination for NES, approaching chance in some cases ^2^. Eighty-five percent of subjects in the study reported a “formal (certain or highly likely) diagnosis of NES” but it is not clear that these subjects were diagnosed in accordance with ILAE standards.

 Relevant text and additional citations added to the first paragraph of the Strengths & limitations section.

 2) Subjects were recruited through advertisements to NES support groups to participate in an anonymous online survey. There was no verification that respondents actually met the limited inclusion criteria described above. The authors acknowledge this limitation […] Additionally, the diagnosis of NES is notoriously complex and difficult, subject to confusion and error at many steps. As such there is a legitimate question as to how many subjects actually had NES.

 As per above, text and citations added to the first paragraph of the Strengths & limitations section.

 They argue “recent reviews suggest health data can be collected with equal or… better reliability in Web-based questionnaires compared to traditional approaches.” Citations should be given here.

 Apologies. Text elaborated on and citations added.

 3) Similarly there could be no verification of subjects’ reported experiences. While some of the reported interactions are very plausible, others sound like the subjects may be interpreting or elaborating dismissive but less dramatically offensive behavior on the part of a provider (“he laughed in my face at the diagnosis of FND,” “I can remember her just standing over me… shouting ‘get up you are wasting my time, why do I have to put up with patients like you’”). There is great value in these reports because they illustrate the subjects’ perceptions and suffering, but it’s questionable whether they should be interpreted literally. The authors generally discuss the responses as illustrations of patients’ perceptions but on occasion appear to interpret reports literally (“participants recount practitioner conduct that defies all ethical and professional obligations and standards; and which has not been previously reported in the literature to our knowledge”).

 We have added some text to the methods (analytical approach) section to clarify our position with regard to interpretation of participants’ reported experiences. Presented here for ease: “The purpose of the data analysis was to seek an interpretative understanding of participant’s stories (Weber, 1922), which means that they “hold truth in the sense that they allow us to understand reality from the point of view of the person expressing these comments” (De Maio, 2010, p.150). In line with this perspective, we do not question whether expressed experiences are right or wrong, good or bad”.

 Regarding the language used when interpreting participants texts, this ties into Pia Bülow’s comment, point three: so we address both together. The original wording in the discussion reflected analysing participant experiences according to their overarching narrative. We have edited relevant text and included words such as “perceive”, “depict”, “relate”, “recount”, etc.

 4) There is a (perhaps understandable) lack of attention to the motivations and subjective experiences of the providers involved in the reported interactions. Some of the most disturbing quotations describe emergency medicine (EM) physicians apparently physically torturing subjects, and then crowing that the subjects are “faking” when they respond to physical discomfort (“He stabbed my arms with a needle whilst I was paralyzed to prove I was faking…. He kept telling me I was faking”). The context behind such encounters is that EM physicians need to determine within 5 minutes whether a patient with ongoing seizure activity is in status epilepticus (a life-threatening condition which should be aggressively treated with potentially dangerous sedative medications, possibly leading to intubation and iatrogenic injury or death) or NES (which should not be treated with medications). Unlike neurologists in epilepsy monitoring units, EM physicians must usually make this determination without the aid of EEG monitoring. As a result they often resort to noxious stimuli (sternal rub, nailbed pressure) in an attempt to get an accurate measure of responsiveness. While stabbing a patient with a needle is almost certainly unnecessary (and a diagnosis of NES should not be equated with faking), such behavior is often motivated by concern for the patient’s life rather than a desire to trick the patient. It is invaluable for physicians to understand how patients perceive this behavior, but it also important for patients and researchers to understand the perceptions and motivations of healthcare providers. Increasing communication between patients and providers and between disciplines, as exemplified by this study, may aid in both goals.

 We agree that EM staff are under a great deal of pressure to determine the nature of a seizure, and for very good reason. However, what is striking about our participants’ accounts in this context (as you picked up on) are the accompanied accusations of “faking” (and implied voluntary control). Indeed, the vast majority of related accounts include participants recalling being told they were “faking”. Notably, in our data some patients describe stimuli in acute contexts using ‘technical terms’, e.g. “sternum rub” and “pin prick test”; and appear aware of the need for and motivations behind these tests. What seems to agitate them is the accompanied accusations. There are then other accounts of ‘stimuli’ beyond the realm of medical necessity (“bent a pillow around my face”, “head hit”, etc.) As a side, we would also like to add that in terms of the extracts presented, we purposefully excluded some of the most dramatic to avoid appearing overly sensationalistic. So while we agree it is important that patients and researchers understand practitioner motivations in acute contexts (and we have added text to the discussion, bottom of “execution of disrespect section” to this affect), we believe the most important message here (for us to convey) is for health practitioners at the front line to reflect on the moral judgements they convey, and thoughtfully consider the potential consequences of their actions. In short, to do what is medically necessary but put an end to any moral blaming and accusations of “faking”.

 *5) The lack of a control group raises an important question: how different -- how much worse – were the experiences of the subjects in comparison to the experiences of patients with other chronic illnesses? Were researchers to prompt groups of patients with other chronic illnesses to think of their worst healthcare experiences, it seems likely that many would recount truly horrific experiences. A control group with a different chronic illness would add a great deal to the interpretation of the subjects’ responses, and failing that a direct comparison by the authors to similar web surveys of different patient groups would be helpful.

 This ties in with a comment made by Prof. Dowrick, and so we address both together. We have reflected on the possible similarities/differences between the negative experiences of people with NES and those reported by people with other MUPS, and have dedicated a paragraph to this in the Strengths and limitations section. We have now explicitly pointed out that the lack of a comparison group is a limitation of this study.

 *6) The respondents to the survey are very likely biased toward those with more negative experiences, whereas those without intense negative experiences would presumably have less motivation to complete a lengthy and uncompensated online survey. The authors appropriately identify this likely bias as a limitation.

 Although not appearing to require action, we agree, and have changed “possible bias” to “likely bias”.

# Peer review: Referee response for version 1

**DOI:** 10.21956/wellcomeopenres.13132.r24462

**Published:** 2017-07-25

**Authors:** Christopher Dowrick

**Affiliations:** 1 Institute of Psychology Health and Society, University of Liverpool, Liverpool, UK

This is a valuable contribution to the limited literature on the experience of patients with non-epileptic seizures (NES) in their encounters with health care professionals - encounters which are known to often be a source of mutual frustration. The authors explore data provided by 135 respondents to an online survey about NES, focusing on responses to three question related to the respondent's 'single worst interaction' with a health professional. Taking a thematic discourse analytic approach, they identify six key themes, including lack of knowledge and respect, disregard of experience and patient-blaming, and conclude that these encounters are typified by negative normative judgements on the part of health professionals, leading to a sense of humiliation and stigma on the part of patients with NES.

 The authors reasonably describe the potential limitations of online survey responses, including self-selection biases, and note that most respondents were women from UK or USA. As balance, they note emerging evidence for the utility of online surveys, including allowing respondents time for reflection.  They also explain why the confidential nature of the survey restrict their ability to offer all source material for independent scrutiny.

 The authors' findings and conclusions have authenticity. They should provide useful material - inter alia - for education and training of health professionals, especially for those working in accident and emergency departments who are the most common professional group involved in these negative encounters.

 The paper could be strengthened in two ways:

1. It would be helpful to know more about the intentions, content and structure of the 86-item survey from which these three questions were drawn, in order to better understand their context. For example, were there also questions about 'single best interaction' or about other positive or neutral experiences? It would also be useful to know what further analyses of the full dataset are being considered or planned.
2. When setting this study in the context of existing literature, the authors could also usefully make reference to the broader literature on health encounters involving contested/medically unexplained symptoms (e.g. Peters *et al*., 2009 ^1^, Dowrick *et al.,* 2008 ^2^).  They might also reflect and comment on whether they consider the negative experiences of patients with NES to be similar to those reported in these commonly distressing encounters, or whether they have important elements which make them unique.

I have read this submission. I believe that I have an appropriate level of expertise to confirm that it is of an acceptable scientific standard, however I have significant reservations, as outlined above.

## References

[1] PetersSRogersASalmonPGaskLDowrickCToweyMCliffordRMorrissR: What do patients choose to tell their doctors? Qualitative analysis of potential barriers to reattributing medically unexplained symptoms. *J Gen Intern Med*.2009;24(4) : 10.1007/s11606-008-0872-x 443-9 10.1007/s11606-008-0872-x 19089505PMC2659146

[2] DowrickCGaskLHughesJGCharles-JonesHHoggJAPetersSSalmonPRogersARMorrissRK: General practitioners' views on reattribution for patients with medically unexplained symptoms: a questionnaire and qualitative study. *BMC Fam Pract*.2008;9: 10.1186/1471-2296-9-46 46 10.1186/1471-2296-9-46 18713473PMC2533666

# Response

**Published:** 2017-09-15

**Authors:** Catherine Robson

**Affiliations:** University of Tromsø, Norway

Dear Professor Dowrick,

 Firstly, our apologies for not responding sooner. The first author has recently moved countries, and has had to attend to a host of family and job matters.

 Thank you for your thoughtful consideration of the paper, and for your constructive comments. For expediency, we will address these after we receive the second peer review. 

 Best wishes,

 Catherine and Olaug.

# Response

**Published:** 2017-10-13

**Authors:** Catherine Robson

**Affiliations:** University of Tromsø, Norway

We would like to thank the reviewers for their insightful comments; which have added more depth, substance … and clarity to this paper. We are especially pleased that reviewers from different disciplines and areas of practice (a GP, neurologist and social scientist) took the time to thoughtfully review our work – and we sincerely thank you. We address the comments in turn below, removing some background text that did not require action. 

**Christopher Dowrick**, Institute of Psychology Health and Society, University of Liverpool, Liverpool, UK  

 1.It would be helpful to know more about the intentions, content and structure of the 86-item survey from which these three questions were drawn, in order to better understand their context. For example, were there also questions about 'single best interaction' or about other positive or neutral experiences? 

 We have added more explanatory text about the overall research project to the Methods section; discussing the overall aims and the final key themes survey data were organised around.

 We briefly mentioned other relevant survey questions (e.g. “single best interaction”) in the Strengths and limitations section, but have elaborated this to make it clearer. This includes adding a reference to a paper that incorporates this data: Lian, O.S. and C. Robson, Socially constructed and structurally conditioned conflicts in territories of medical uncertainty, *Social Theory & Health*, (under review).

 1B.       It would also be useful to know what further analyses of the full dataset are being considered or planned.

 With reference to the response above, we have also added some additional text to the Methods section that explains other aspects of the data we broadly aim to examine.

 For your information (we have not included this in the main text as the paper is running rather long – but hope interested parties can read our response here) further analyses in progress include:

 A statistical analysis of data from (n=115) participants who provided socio-demographic and health-related data and completed a series of questions investigating their HRQoL (QOLIE-31) and stigma perceptions (10-item Epilepsy Stigma Scale). The paper, “Health related quality of life of people with non-epileptic seizures: the role of socio-demographic characteristics and stigma”, is currently under review *(Seizure).*

 Two postgraduate (Clinical Psychology) students at the University of Pretoria, South Africa are using data to explore:

 Dissertation title: The perceived effect of different treatment approaches on the health related quality of life (HRQoL) of patients with psychogenic non-epileptic seizures (PNES). Research question: What is the perceived effect of different treatment approaches on the HRQoL (measured using the QUOLIE-31) of patients with PNES?

 Dissertation title: The Perceived Impact of Different Treatment Approaches on Stigma in Patients with Psychogenic Non-Epileptic Seizures (PNES) Based on Data Collected from Online Support Groups in America, Australia, and Europe. Research Question: What is the perceived impact of different treatment approaches on stigma (measured using the epilepsy stigma scale) in patients with PNES?

 The first author (and possibly the second) also plan two further papers from the data:

  

1. A mixed method study exploring the diagnostic journey, with a focus on the presentation and acceptability of aetiological explanations.
2. A qualitative study examining responses to questions asked under the ‘social support and social stigma’ theme (focus undecided at present).

2.When setting this study in the context of existing literature, the authors could also usefully make reference to the broader literature on health encounters involving contested/medically unexplained symptoms (e.g. Peters *et al*., 2009, Dowrick *et al.,* 2008). 

 2B.       They might also reflect and comment on whether they consider the negative experiences of patients with NES to be similar to those reported in these commonly distressing encounters, or whether they have important elements which make them unique.

 We have reflected on the possible similarities/differences between the negative experiences of people with NES and those reported by people with other MUPS (including the references you mention), and have dedicated a paragraph to this in the Strengths and limitations section.
